# Study of Burn Scar Extraction Automatically Based on Level Set Method using Remote Sensing Data

**DOI:** 10.1371/journal.pone.0087480

**Published:** 2014-02-04

**Authors:** Yang Liu, Qin Dai, JianBo Liu, ShiBin Liu, Jin Yang

**Affiliations:** 1 Institute of Remote Sensing and Digital Earth, Chinese Academy of Sciences, Beijinng, China; 2 University of Chinese Academy of Sciences, Beijing, China; Leiden University Medical Center, Netherlands

## Abstract

Burn scar extraction using remote sensing data is an efficient way to precisely evaluate burn area and measure vegetation recovery. Traditional burn scar extraction methodologies have no well effect on burn scar image with blurred and irregular edges. To address these issues, this paper proposes an automatic method to extract burn scar based on Level Set Method (LSM). This method utilizes the advantages of the different features in remote sensing images, as well as considers the practical needs of extracting the burn scar rapidly and automatically. This approach integrates Change Vector Analysis (CVA), Normalized Difference Vegetation Index (NDVI) and the Normalized Burn Ratio (NBR) to obtain difference image and modifies conventional Level Set Method Chan-Vese (C-V) model with a new initial curve which results from a binary image applying K-means method on fitting errors of two near-infrared band images. Landsat 5 TM and Landsat 8 OLI data sets are used to validate the proposed method. Comparison with conventional C-V model, OSTU algorithm, Fuzzy C-mean (FCM) algorithm are made to show that the proposed approach can extract the outline curve of fire burn scar effectively and exactly. The method has higher extraction accuracy and less algorithm complexity than that of the conventional C-V model.

## Introduction

Burn scar refers to areas that are destroyed by forest fire, grass fire and controlled burning and have not yet recovered. Forest fire is one of the most severe natural hazards. It impacts ecology structure, atmospheric systems, as well as having detremental effects on living environment. For these reasons, in order to decrease the effect of forest fire, how to detect active fire and evaluate burn area rapidly with high accuracy has to be settled urgently [1]. Detecting and assessing the spatial extent and distribution of burn scar can support forestry services to process efficient vegetation recovery and post-fire management. In general, burn area has the properties of large size and spatial variability. Remote sensing has the advantages of wide viewing angles, multi-spectral imaging and multi-temporal revisit. It has become a primary tool for extracting burn scar. The destroyed landscapes caused by forest fire are observed by satellites from space at different scales is becoming the focus of researchers across the global. Over the past several decades, a wide variety of satellite datasets have been used to generate burn scar, such as the National Oceanic and Atmospheric Administration Advanced Very High Resolution Radiometer (NOAA/AVHRR), SPOT VEGETATION, Along-Track Scanning Radiometer (ATSR), Moderate Resolution Imaging Spectroradiometer (MODIS), Landsat Thematic Mapper (TM) and the Enhanced TM plus (ETM+) [2]–[4]. Besides, the Landsat 8 satellite launched on February 11, 2013. It will replace Landsat 5 to acquire valuable data and imagery to be used in agriculture, education, business and politics. Furthermore, it becomes another data source for extracting burn scar.

Today, many techniques have been developed to derive burn scar information from remote sensing data: fixed thresholding algorithm with multi-spectral images or indices computed from pre-fire and post-fire images and post-classification techniques using multi-temporal data [5]. These methodologies have been commonly applied to specific fire events. However, very few of them can comprehensively adapt to as many aspects as possible. The fixed thresholding algorithm discriminates burn scar from neighbouring objects with empirically derived thresholds [6]. Researchers have defined a set of fixed thresholds to extract burn scar. This method has advantages of simplicity and processing speed, whereas the limitation is that adequate thresholds related to various reasons are difficult to choose. In order to overcome this limitation, researchers have developed automatic threshold methods based on mean value and Standard Deviation (SD). Fernandez *et al.* (1997) propose a method to obtain thresholds automatically which is defined as mean+2*SD from a NDVI differencing image within local window [7]. Barbosa *et al.* (1999) present the V13T threshold method as the mean-SD for each pixel with long temporal series [8]. However, Vafeidis and Darke (2005) find that the above methods based on SD would not get well results for different cases. Therefore, the fixed thresholds algorithms still face the challenges to choose the optimal thresholds to extract burn scar in an automatic fashion [9].

Post classification techniques use image data and indices derived from multi-temporal images to make image classification and compare the post-classification image to extract burn scar. For example, X. Cao *et al.* (2009) combine the GEMI-B and SVMs classifier to extract burn scar in grassland areas with high accuracy [10]. Pereira *et al.* (1999) use classification and decision trees to segment NOAA-AVHRR imageries into burn surface, unburn surface, and clouds [11]. The result of this method matches well with the ground truth. However, extraction accuracy of algorithms above depends on the training data of classification. In fact, the ground truth samples data are difficult to obtain.

Despite the simplicity and widespread use of the above mentioned algorithms, there exists a major drawback: lack of an automatic and unsupervised technique to extract burn scar. The objective of this study is to develop and test an unsupervised method to automatically extract burn scar without predefined thresholds. The proposed method includes two-step process. First, compare the bi-temporal images which are taken over the same geographical area at different dates. “difference image” is produced by the comparison between pre-fire and post-fire images. Methods of obtaining difference image is generally divided into three categories: (1) simple algebraic method, for example: image differencing, image ratioing, regression analysis, etc [12]. (2) based on transformation: Principal Component Analysis (PCA), Change Vector Analysis (CVA), etc. (3) based on image features: texture, gradient, vegetation indices and so on. The methods mentioned above have made a difference on the specific aspect. However, these ones only utilize single feature to get the result, sometimes leading the extraction results are inaccurate and incomplete. Consequently, this paper proposes a method that creates the difference image via combining weighted features.

After the first step, separating the burn scar from difference image is a significant job. According to the characteristics of burn scar, this paper focuses on developing an automatic procedure to extract burn scar based on Level Set Method (LSM) without pre-defined information. LSM was proposed by Osher and Sethian in 1988, which was applied on the hydrodynamics problems [13]. LSM expresses loop curve as zeros level set of three dimensional continuous surface. It transforms the process of solving curve function into a partial differential equation of zeros level set. LSM takes it easy to follow shapes that changes topology, such as splitting, merging and developing holes. Then it has played an important part in wide fields such as: physics, materials and computer vision, etc. LSM consists two parts based on edge and region. Caselles *et al.* (1993) put forward the Geodesic Active Contour (GAC) model which is based on edge [14]. Kimmel (2003) modifies the GAC model with joining the direction information of edge [15]. Nevertheless, when the imagery is vague, edge-based model does not perform very well. Moreover, if the objects’ shapes within the image are sunken, the curve may not shrink inward leading the phenomenon that the final position of curve does not coincide with the object’s real boundary. On the contrary, the model integrating global gray information behaves well, which can deal with the imagery with blurred and sunken borders. Mumford and Shah (1989) come up with the Mumford-Shah model to segment image by minimizing a energy funciton [16]. Chan and Vese (2001) utilize LSM to refine the Mumford-Shah model to simplify the process of solution, called C-V model [17]. C-V model uses the gray-value feature not gradient to partition the imagery into background and object parts. Remote sensing imagery of burn scar has various shapes caused by wind or terrain, so the boundary may be blurred and sunken. Thus, C-V model is adapted to extract burn scar. Furthermore, setting initial contour makes some effect on iteration numbers when applying C-V model to segment images. How to set initial curves of C-V model influences the speed and accuracy of extraction. There are many researchers have presented strategies to set effective initial contour. Hichiri (2013) proposes to consider the result of Support Vector Machine classification [18]. Cao *et al.* (2007) uses binary imagery of interested objects as an initial curve via selecting areas interactively and morphological algorithm [19]. This paper also presents new method to modify the initial curve of LSM to reduce the time of consuming and improve the precision of burn scar extraction.

In summary, the proposed method can overcome the disadvantages of traditional methods with selecting empirical thresholds and depending on training set. The novel method takes advantage of LSM and multiple features to extract burn scar quickly and automatically.

## Materials and Methods

### Study Area and Data

**Figure 1:**
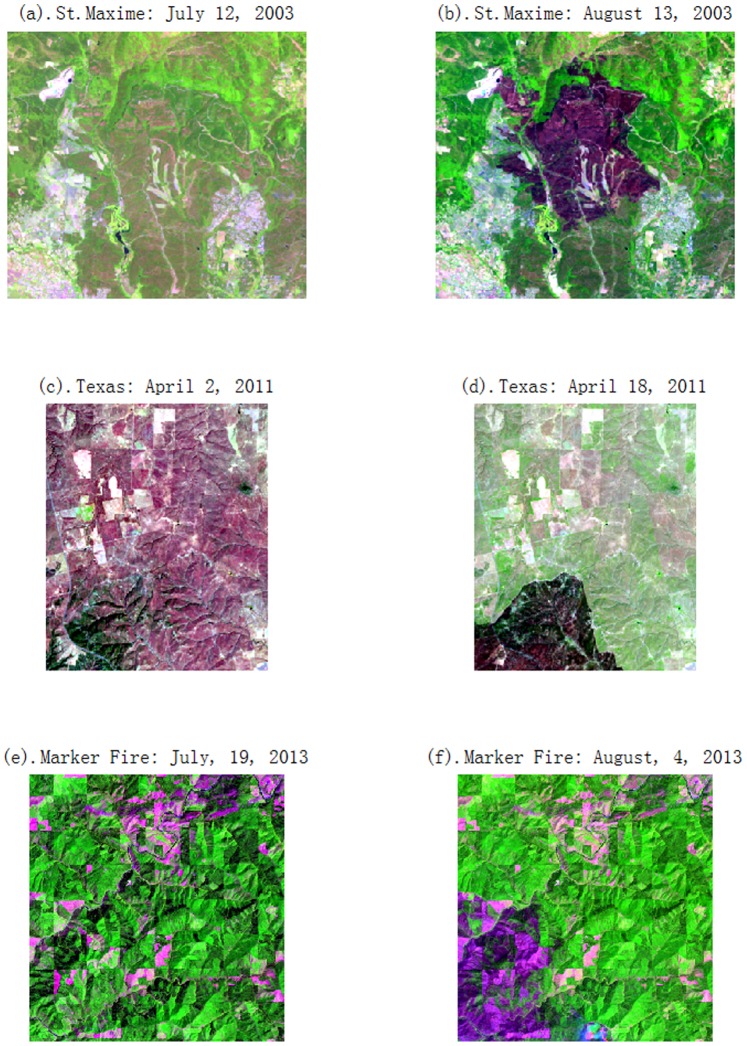
False color images of three data sets. (a). St. Maxime image of July 12, 2003; (b). St. Maxime image of August 13, 2003; (c). Texas image of April 2, 2011; (d). Texas image of April 18, 2011; (e). Marker Fire image of July, 19, 2013; (f). Marker Fire image of August, 4, 2013.

1. The first data set used in the experiments consists of two co-registered images (356×317 pixels) taken near St. Maxime, France, located between the longitudes of 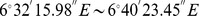, and latitudes of 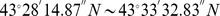, on the Mediterranean coast by Landsat 5 Thematic Mapper (TM) in the July 12 and August 13, 2003, respectively. During the two acquired dates, a fast-moving forest fire occurred on July 28, 2003, destroyed nearly 16,000 acres of woodland. Fig. 1(a) and (b) show false color images composited with 5,4,3 bands, respectively.
2. The second data set are two co-registered images of 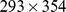 pixels acquired by Landsat 5 Thematic Mapper (TM) on the April 2, 2011 and April 18, 2011, respectively. This fire occurred on April 9, 2011 in the central Texas, USA located between the longitudes of 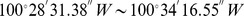, and the latitudes of 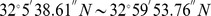. The false color images composited by 5,4,3 bands are showed in Fig. 1(c) and (d).
3. The area located in 15 miles Northeast of Goldendate, Washington is selected as third data set. The data set is captured by Operational Land Imager (OLI) on Landsat 8 before and after the Mile 28 Marker fire. Mile 28 Marker fire occurred in late-July, 2013 and destroyed 10,220 acres in Washington. The subset of this data set with 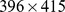 pixels locates between 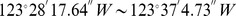, and the latitudes of 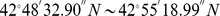. Fig. 1(e) and (f) show the false color images composited by 6,5,4 bands taken on July, 19, 2013 and August, 4, 2013.
4. The experiment environment is Matlab R2010b platform based on windows 7 system with 8G memory.

In the view of Fig. 1(a)–(f) images, it can be seen that the shapes of burn scar are variable and the boundary may be sunken even blurred. Traditional methods sometimes have difficulty in discerning the burn scar exactly and completely. In the Results section, the proposed method can be validated that it can extract complete and clear burn scar.

### Burn Scar Extraction Automatically Based on Level Set Method

**Figure 2:**
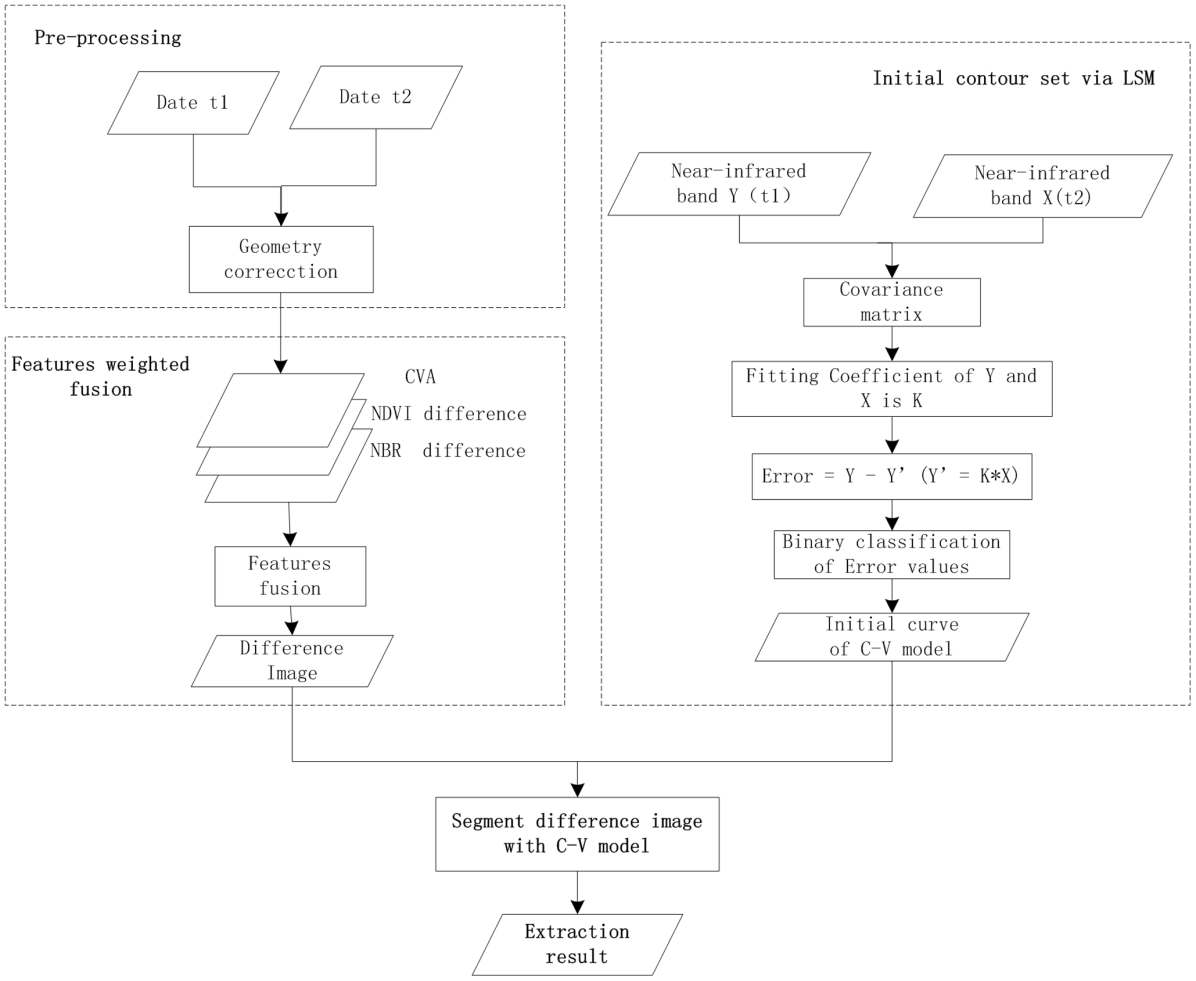
Procedure of fire burn scar extraction based on LSM.

The proposed technique flowchart is shown in the Fig. 2. The whole process is divided into three parts: First, integrate the weighted spectral features CVA, NDVI and NBR difference of the Date 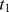 and Date 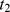 which are corrected geometrically to get the difference image. “Features weighted fusion” sub-section introduces this step. Then, analyze the linear fitting error of two near-infrared band images which are got at 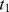 and 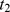 respectively and refine the initial curves through binary classification result acquired by K-means method. The second step is described in details in the sub-section “Initial contour set of LSM”. Finally, make the difference image and initial contour gained above as the input and the output is extraction result of burn scar. The sub-section “Segment difference image with C-V model” presents how to process the last step of proposed method.

### Features Weighted Fusion

Traditional extraction method based on LSM segments the difference image obtained by CVA methodology to extract burn area information. Considering the fact that NDVI and NBR indices change obviously because fire wrecks the vegetation cover of forest [4]. NBR has been widely used to discriminate burn area from unburn area [20]. NDVI is claimed to solve the confusion between classes in the remote sensing images better than NBR [21]. This paper aims at integrating CVA, NDVI difference and NBR difference to get the final difference image. Like this, the difference image can enhance the recognition characteristics of burn scar.

The principle of CVA is describing the difference of single feature across the different bands between two dates. The difference represents the change of pixels in individual band. The results calculated by CVA include magnitude and direction parts. Basically, a change vector can be described with variables of each band, and magnitude component expressed the amount of the change as the direction component. Assuming that 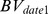 and 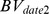 are pre-fire and post-fire geometrically coregistered remote sensing images with 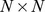 pixels and 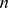 bands, which are taken from the same area at different dates. 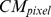 represents the pixel value of the difference image by applying CVA algorithm. 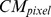 is given by (1)[22]:
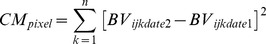
(1)where 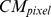 describes spectral difference of two different remote sensing images. The larger the amount of 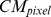 is, the higher the probability of change occurs. 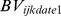 and 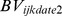 are the 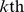 band value of pixel located at 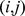 corresponding to Date 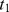 and Date 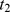 images respectively. 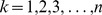, 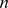 is the number of remote sensing image bands.

The NDVI is calculated as follows:
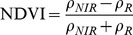
(2)where 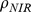 and 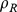 stand for the reflectance acquired in the near-infrared and visible (red) bands respectively. Digital number values of remote sensing images are used instead of reflectance in this paper. The NDVI difference is given by:
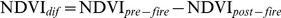
(3)NBR is proposed by Key and Benson in 2004 [23]. They replace the red reflectance in the NDVI with the mid-infrared reflectance value. The mid-infrared reflectance is sensitive to water of vegetation and the lignose content of non-photosynthetic vegetation. The index is given below [24]:
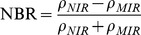
(4)where 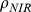, 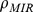 are reflectance in the near-infrared and mid-infrared bands, respectively. The NBR difference is described below:



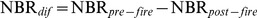
(5)In order to utilize the advantages of multiple features, this paper uses Chi Square Transformation (CST) algorithm to weighted fuse the normalized difference image obtained from CVA, NDVI and NBR algorithms to create difference image between pre-fire and post-fire data. The weight values are obtained by the standard deviation of each difference image component [25].
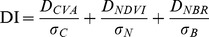
(6)


In (6), DI is the weighted difference Image, 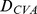 is the difference image acquired according to CVA algorithm, 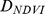 is the difference image calculated by NDVI feature and 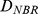 is the difference image obtained by NBR feature. Normalize the DI which is the final difference image by fusing the distinct difference images, where the coefficients 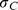, 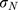 and 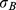 are the standard deviation of three difference images, respectively.

**Figure 3:**
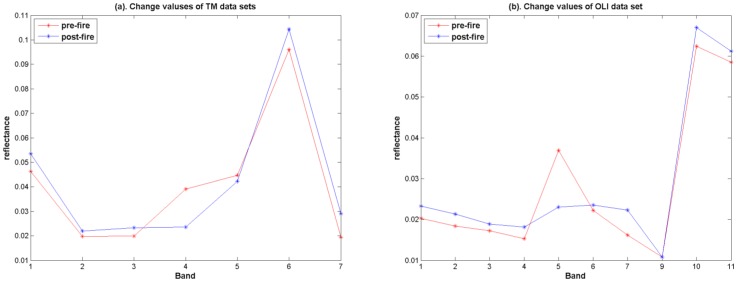
Change values of different bands of TM and OLI data between pre-fire and post-fire. (a). TM data sets; (b). OLI data sets.

After the fire, the reflectance of burn scar area will have a change at different bands. The change values of selected bands data of TM and OLI images within burn scar area are shown in Fig. 3.

Fig. 3 shows that reflectance values of all bands have changed after the fire. The change values of red, near-infrared and mid-infrared bands are obvious, especially the near-infrared band. NDVI is calculated by combining red and near-infrared bands data. NBR combines near-infrared band and mid-infrared band to estimate the vegetation area effected by fire. CVA represents the difference between pre-fire and post-fire images of all bands in global. At the same time, NDVI and NBR difference can emphasize the difference of red, near-infrared and mid-infrared bands in local. Thus, in order to enhance the burn scar reflectance features, the weighted fusion algorithm is a better choice.

To illustrate the fusion results, this paper uses the normalized distances 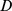 calculated with mean and standard values of burn and unburn area to compare the CVA image and weighted fusion difference image [9].
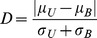
(7)where 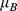 and 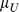 are the means values of burn and unburn areas, 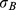 and 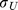 represent the standard deviation of burn and unburn areas, respectively.

The 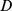 values calculated by CVA and Fusion methods are shown in Table 1.

**Table 1:** D values produced by CVA and Fusion features method.

	CVA	Fusion Methods
TM data (St. Maxim)	1.2565	1.8350
OLI data (Marker Fire)	0.9193	1.5354

CVA represents Change Vector Analysis.

**Figure 4:**
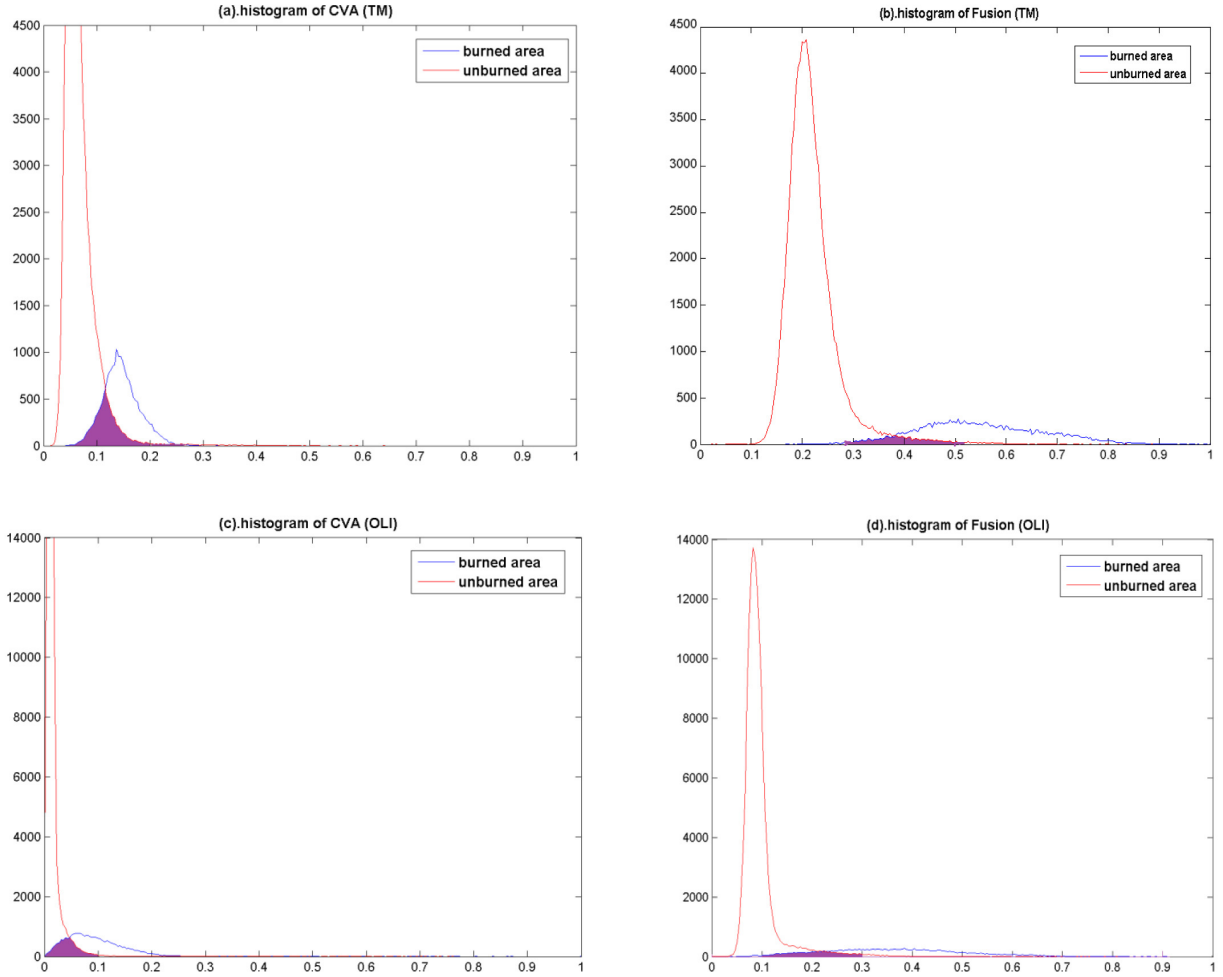
Histogram of burn and unburn class for two data sets. (a). Histogram of CVA :St. Maxime; (b). Histogram of fusion:St. Maxime; (c). Histogram of CVA:Marker Fire; (d). Histogram of fusion:Marker Fire.

According to the Table 1, the 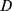 values of difference image acquired via weighted fusion method are higher 0.5785 and 0.6161 than CVA method for TM and OLI data sets, respectively. The higher 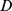 values demonstrate the method can make better discrimination between burn and unburn areas than others. [9] mentions that the larger 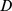 values can indicate low degree of histogram overlap between burn and unburn classes. The histograms of burn and unburn area for TM and OLI data sets are shown in Fig. 4.

Fig. 4 presents that the overlap area between burn and unburn classes produced by weighted fusion features is smaller than one produced by CVA algorithms for TM and OLI data sets. This comparison outcomes can illustrate the fusion algorithm can describe the characteristics of the burn class in the difference image better. Thus, this step provides a guarantee for segment algorithm to discriminate the burn scar from unburn area exactly.

### Initial Contour Set of LSM

Because of the high correlation of pre-fire and post-fire remote sensing images taken by the same sensor from the same area, identification of the burn area can be made by least square method for two acquisitions images. Burn scar belongs to the change information which are within the part with large fitting error values. In order to decide the threshold to separate the Error into “change” and “no change”, K-means algorithm is adopted to analyze the Error values. Then, the binary classification result is as the initial curve. The initial contour set by this method can locate appropriately the boundary of real burn scar, and improve the sufficiency of initial curve.

Suppose 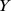 is the 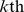 band of date 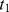 of 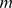 dimensional vector, 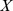 is the 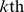 band of date 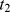 of 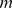 dimensional vector.
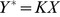
(8)where 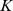 is calculated by least square method, which satisfies the equitation 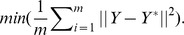 According to this constrained condition the following fitting coefficient can be got:
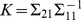
(9)where 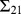 indicates covariance between 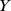 and 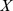, 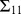 is variance of 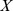.

Error value between 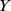 and 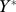 in the following equation is calculated by Mahalanobis distance,
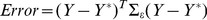
(10)


In (10), 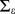 represents the variance of 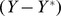, the area with lager 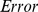 value is defined as the change areas.

### Segment Difference Image with C-V Model

The objective of Mumford-Shah segment method is to find a curve C which can minimize the fitting energy to divide the image into two non overlapping parts. The fitting energy function is:
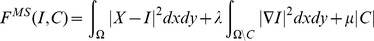
(11)where 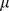 and 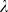 are the parameters selected by users to fit a particular class of images, 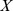 is the image to be segmented, and 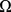 is the defined domain of that image. 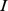 is the average of the region separated by curve 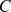.

In fact, it is not easy to minimize the fitting energy function because it is a non-convex surface. Chan and Vese raise to exploit LSM to solve the aforementioned problem. The C-V model simplifies the Mumford-Shah method and the Heaviside function is introduced. This algorithm divides the image into inner region 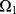 and external region 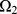 by the curve 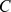. The average values of 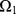 and 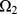 can reflect the difference values of object and background. In this case, the simplified energy function is as follows:
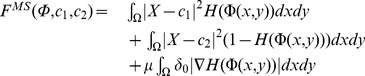
(12)


In (12), 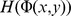 is Heaviside function, 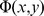 is level set function, 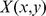 is the image defined in the 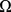, 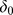 is Dirac delta function 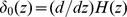. Heaviside function is given by:
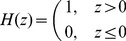
(13)


In practice, Heaviside is replaced by the following regularized format generally:
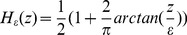
(14)

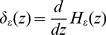
(15)


In (12), 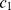 and 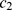 are given by:
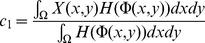
(16)

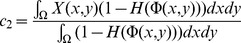
(17)


In (16) and (17), 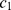 and 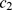 are the average values of inner and external contour 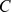 respectively. After the 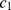 and 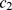 are fixed, Euler-Lagrange equations and the gradient-descent method are used to derive 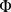 which minimizes the fitting energy function (12):
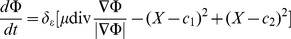
(18)



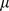
 is regularization parameter set by user, 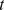 is an artificial time, the solution of (18) is calculated by spatial finite difference method.

## Results

### Set the Initial Contour with Least Square Fitting

**Figure 5:**
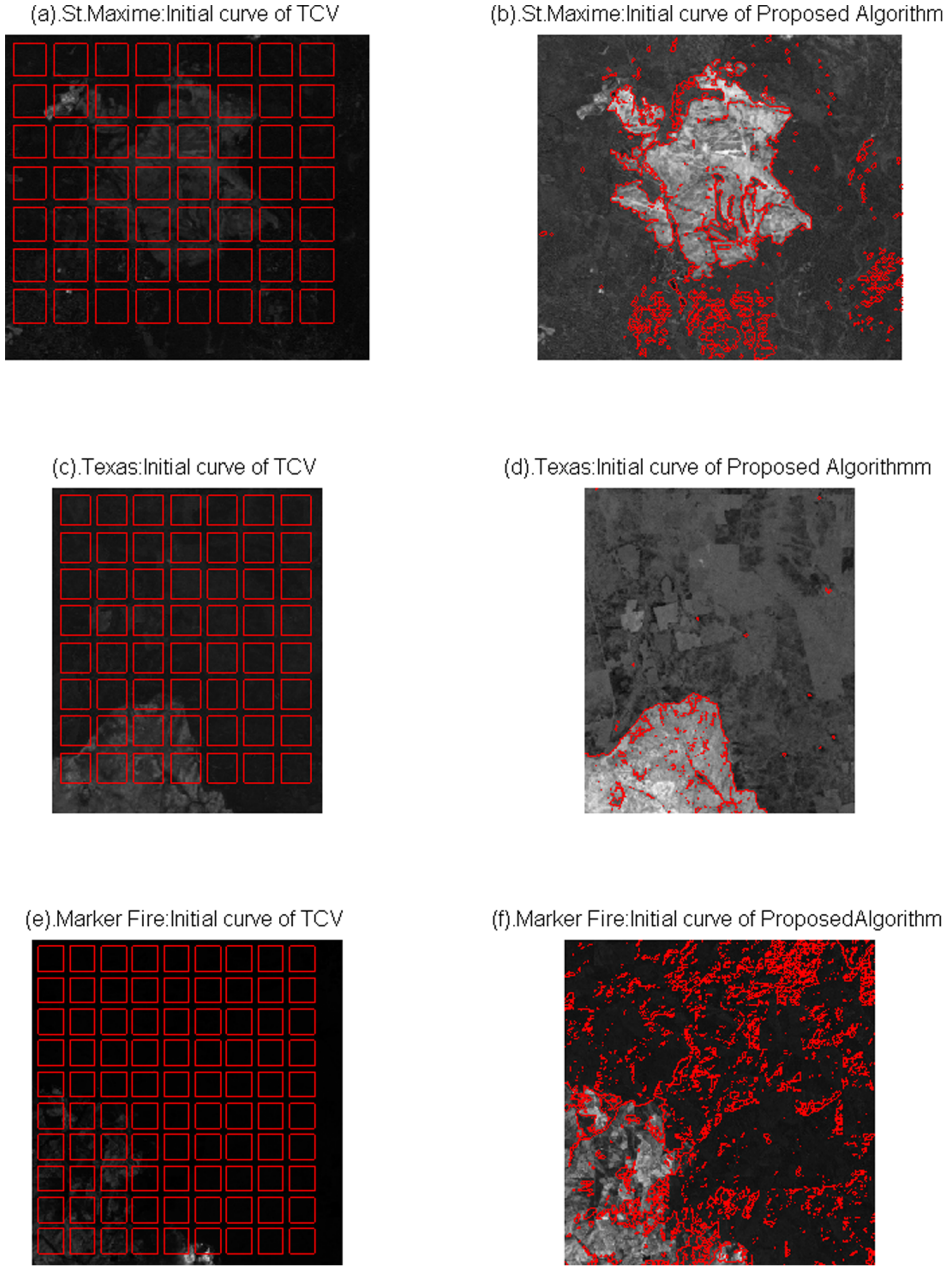
Comparison of different initial curves of three data sets. (a). St. Maxime:Initial curve of TCV; (b). St. Maxime:Initial curve of Proposed Algorithm; (c). Texas: Initial curve of TCV; (d). Texas:Initial curve of Proposed Algorithm; (e). Marker Fire: Initial curve of TCV; (f). Marker Fire:Initial curve of Proposed Algorithm.

The bi-temporal images are observed from same sensor and same area at different time. Due to the evident change of near-infrared band of remote sensing images, it is chosen as the fitting images with least square fitting algorithm. The initial contour of traditional C-V model is arbitrary shapes. Here we choose rectangles filled in the whole image as traditional initial curve. This initial curve is as shown in Fig. 5(a)(c)(e). The initial curve proposed by this paper is as shown in Fig. 5(b)(d)(f). In the view of Fig. 5, the proposed initial curve matches the ground truth a lot, which indicates that the proposed algorithm is more reliable.

### Extraction Results and Analysis

Get difference image of bi-temporal remote sensing images and modify C-V initial curve according to sections above, and then utilize the C-V model to segment the difference image with a new initial contour to extract burn scar.

**Figure 6:**
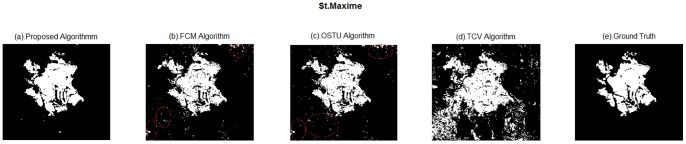
Extraction results of different methods of St. Maxime. (a). Proposed algorithm; (b). FCM algorithm; (c). OSTU algorithm; (d). TCV algorithm; (e). Ground Truth.

**Figure 7:**
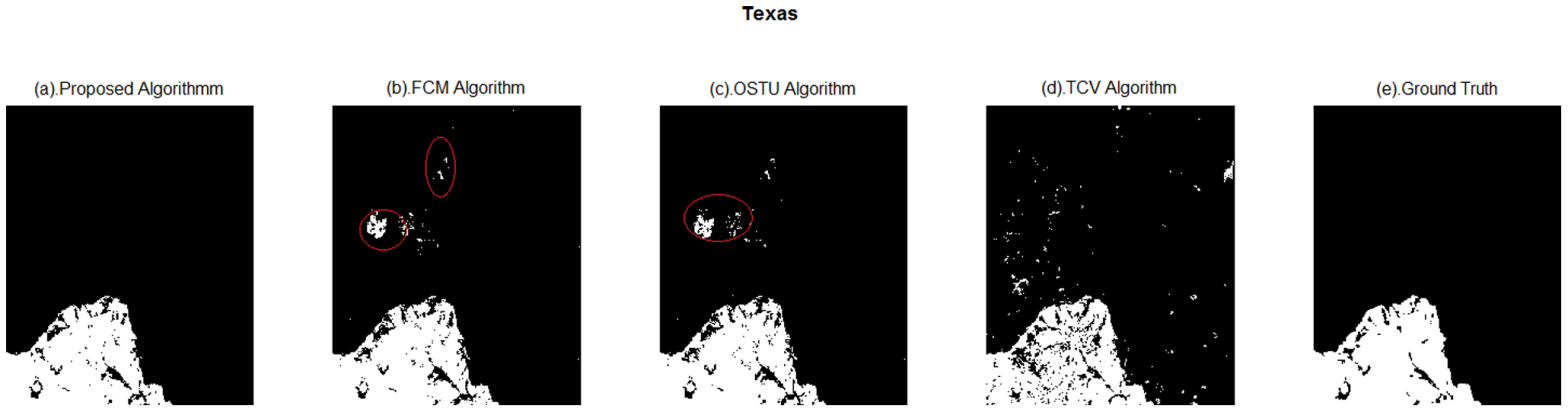
Extraction results of different methods of Texas. (a). Proposed algorithm; (b). FCM algorithm; (c). OSTU algorithm; (d). TCV algorithm; (e). Ground Truth.

**Figure 8:**
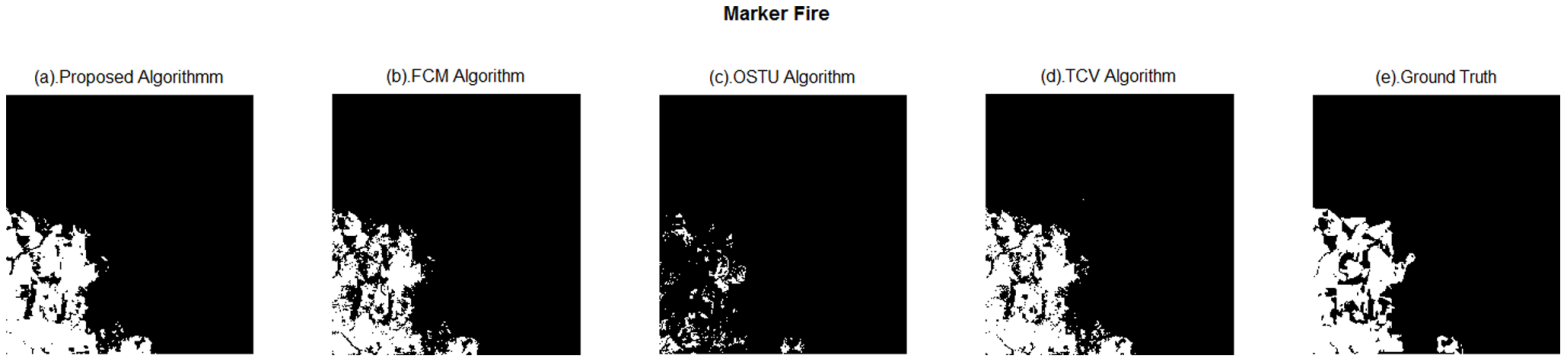
Extraction results of different methods of Marker Fire. (a). Proposed algorithm; (b). FCM algorithm; (c). OSTU algorithm; (d). TCV algorithm; (e). Ground Truth.

To assess the validity of the proposed algorithm, different algorithms are compared with the proposed method: OSTU algorithm, Fuzzy C-mean algorithm (FCM), Traditional C-V model algorithm with rectangles as the initial curves (TCV model). These algorithms are shown in the Table 2. It can be seen from Table 2, the input difference images of OSTU algorithm, FCM algorithm and proposed algorithm are the same. Like this, the extraction results can show the different segment effect of OSTU, FCM algorithms and proposed method. The proposed algorithm has two improvements compared with traditional C-V model, which are methods of obtaining difference image and setting the initial curve. Thus in order to illustrate the effectiveness of the proposed algorithm, comparing it with the traditional C-V algorithm based on two improved points. The results figures of above methods are shown in Fig. 6, 7 and 8.

**Table 2:** Compositing of different methods.

Extraction Algorithm	Difference image	Segmentation algorithm
Proposed algorithm	Fusion results	C-V model with modified initial curve
OSTU algorithm	Fusion results	OSTU algorithm
FCM algorithm	Fusion results	FCM algorithm
TCV algorithm	CVA	C-V model with arbitrary shapes as initial curve

C-V model: Chan-Vese model; FCM: Fuzzy C-mean algorithm; TCV: Traditional C-V model algorithm with arbitrary shapes as the initial curves.

In order to evaluate the availability of the proposed method, this section aims at analysing quantitatively the extraction results with following measures: (1). Missed alarm rate: number of burn pixels classified as unburn pixels; (2). False alarm rate: number of unburn pixels classified as burn pixels; (3). Right alarm rate: the number of burn pixels and unburn pixels classified correctly and (4). Kappa coefficient. Besides, the paper also considers the iteration numbers of proposed algorithm and TCV algorithm. The overall analysis statistics of three data sets as shown in Table 3, 4 and 5.

**Table 3:** St. Maxim: Comparison of different methods in precision of burn scar detection.

Method	Missed alarm rate (%)	False alarm rate (%)	Right alarm rate (%)	Kappa	Iteration
Proposed algorithm	0.90	1.40	97.70	0.9684	349
TCV algorithm	1.59	14.09	84.32	0.7563	2777
OSTU algorithm	1.56	2.95	95.49	0.9372	/
FCM algorithm	2.92	1.58	95.50	0.9374	/

**Table 4:** Texas: Comparison of different methods in precision of burn scar extraction.

Method	Missed alarm rate (%)	False alarm rate (%)	Right alarm rate (%)	Kappa	Iteration
Proposed algorithm	1.06	0.32	98.62	0.9817	196
TCV algorithm	1.91	1.12	96.97	0.9596	3297
OSTU algorithm	1.43	0.77	97.80	0.9707	/
FCM algorithm	1.37	0.82	97.81	0.9708	/

**Table 5:** Marker Fire: Comparison of different methods in precision of burn scar extraction.

Method	Missed alarm rate (%)	False alarm rate (%)	Right alarm rate (%)	Kappa	Iteration
Proposed algorithm	0.65	2.47	96.88	0.9589	890
TCV algorithm	2.61	2.79	94.60	0.9389	5800
OSTU algorithm	10.66	0.0164	89.32	0.8746	/
FCM algorithm	3.27	1.54	95.19	0.9374	/

The detailed analysis of three data sets with different shapes and size are shown as following:

(1) St. Maxime.

In Fig. 6(d), the segment result of TCV algorithm contains a lot of false information, and simply describes the rough boundary of the burn scar. The proposed method is shown in Fig. 6(a), texture of burn scar is more clear, false information is less, and maps with ground truth very well. The information inside the red circles in Fig. 6(b) and Fig. 6(c) show more false boundaries compared with proposed algorithm. Besides, the extraction results of Fig. 6(b) and Fig. 6(c) are scattered. Compared with Fig. 6(e), the proposed method can produce more real extraction results by getting more complete results and reducing false outline.

As seen in Table 3, the kappa value of the proposed method is higher than the other methods obviously for the selected data set. The commission is just 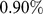, kappa is 0.9684 and the iteration numbers is 349 of the proposed method. The extraction results of OSTU and FCM algorithms are almost the same. Their kappa coefficients are 0.93 nearly with a lower result compared with proposed method. The kappa coefficients of TCV is 0.7563 which is the lower than the other methods. The curve of novel approach can achieve the optimal location quickly because newly developed initial curve setting is close to ground truth outline. From a qualitative point of view, the proposed method can extract the higher accuracy results with least iteration numbers than the other methods.

(2) Texas.

Comparing the Fig. 7(a)–(e), Fig. 7(a) has a close result mapped with ground truth. There is no fire happened in the area inside the red circle of Fig. 7(b) and (c) actually. TCV algorithm results look scattered compared with proposed method.

In the view of Table 4, in terms of kappa coefficients and iteration numbers, proposed methods produce 0.9817 and 196, respectively. Comparing this result with the ones obtained by TCV algorithm, the proposed method shows clear boundary and less false information. This result is corresponding with visually comparing results. OSUT and FCM produce the same result with kappa coefficient is 0.97.

(3) Marker fire.

Fig. 8 illustrates the efficiency of proposed method visually, Fig. 8(a) is confirmed very close to Fig. 8(e) and its outline is clear and complete. By contrast, Fig. 8(d) and Fig. 8(b) have more false information than Fig. 8(a). The OSTU algorithm performs not well with incomplete burn scar results.

Table 5 points out the proposed method can get better extraction results compared with the other ones. The validation results obtained by proposed algorithm are equal to 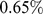, 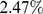, 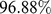, and 0.9589, 890, respectively. TCV algorithm needs to iterate 5800 to meet convergence. However, proposed method decreases the performing time. The results derived by OSTU algorithm is the lowest of all, and the FCM and TCV have the nearly extraction results.

## Discussion and Conclusion

Burn scar area data are important for research on forest recovery and environment change. Multi-temporal remote sensing data is perfect source for burn scar extraction. The existing methods have some limitations. The fixed threshold method depends on the experience. Usually, the adequate threshold is difficult to obtain under various cases. In practice, training set of supervised method cannot satisfy all kinds of burn scar due to their diversity on seasons and spatial extent. Therefore, relying on subjectivity, the training set needs to be increased or adjusted continuously, even interact with human. In order to overcome the limitations mentioned above. This paper proposes an automatic and unsupervised method to extract burn scar in forestry area.

The contributions in this paper are: (1) Weighted fuse the CVA, NDVI and NBR difference to create difference image which makes the burn scar outstanding and contains more rich and accurate information when extracting them. (2) Modify initial contour setting by using least square method between near-infrared images of two dates considering the vegetation cover is destroyed by forest fire. (3) Utilize C-V model to extract burn scar. The C-V model can separate the burn pixels from unburn pixels by handling the topology changes of curve automatically. This step can overcome the disadvantage that the blurred and irregular image boundary is difficult to extract. Besides, it makes the details information of burn scar more clear.

This method has been validated by comparing with TCV, OSTU, FCM methods using Landsat 5 TM data set and Landsat 8 OLI data set. The comparison results demonstrate the proposed approach can achieve better extraction results than the others. Although the three burn scar mentioned above have different shapes and are taken from different data source, the proposed method gets perfect results as well. At the same time, new optimal initial curve reduces the iteration numbers and speeds the extraction of burn scar. The experimental results illustrate that this newly developed algorithm can realize the automatic processing of burn scar extraction with higher accuracy.

This method is suited to be applied on the burn scar extraction of forestry area, especially covered with vegetation. The prerequisites of proposed method are that the two dates images are covered without cloud and taken at the same season. Due to cloud cover, season change may confound extraction results of proposed method. So in the future, in order to extract burn area more conveniently and exactly. This paper would take cloud and time interval factors into account. Through experiments analysis, the results indicate that this method can be easily adapted and applied to extract burn scar from medium resolution satellite images without cloud after the fire occurred. High resolution satellite image is difficult to acquire and has more complex properties than medium ones. Further, the proposed method would get hold of different resolution satellite images into consideration to realize the burn scar extraction of real time satellite data automatically and timely. Once the fire hazards happen, it is always difficult to get the sample data of the disaster areas in a short time for supervised methods. As a better alternative method, the proposed method can obtain extraction results timely and accurately to help the forestry department compute the area destroyed by fire and process vegetation recovery quickly.
